# Monitoring Antibiotic Use in Public Health Care Facilities of South Indian Union Territory: A Step to Promote Rational Use of Antibiotics

**DOI:** 10.7759/cureus.18431

**Published:** 2021-10-01

**Authors:** Dinesh K Meena, Mathaiyan Jayanthi

**Affiliations:** 1 Pharmacology, Jawaharlal Institute of Postgraduate Medical Education and Research, Puducherry, IND

**Keywords:** upper respiratory tract infection, national action plan on antibiotic resistance, rational use of antibiotics, primary health care facilities, antibiotics resistance

## Abstract

Introduction

Antimicrobial resistance is a serious problem to solve for the public health authorities at the global level, particularly in developing countries like India. One of the possible reasons for antimicrobial resistance could be the inappropriate or overuse of antibiotics. The Indian government started the National Action Plan on Antimicrobial Resistance to promote rational use of antibiotics in our country. This study was conducted with the objective to monitor antibiotic use in public health facilities of Puducherry which is a union territory of south India.

Methods

Total 900 prescriptions were prospectively collected from the 10 public health facilities (nine primary health centres and one outpatient department of tertiary care hospital) over the period of one year to analyse antibiotic use.

Results

We found that 36.66 % of prescriptions contained at least one antibiotic. Our result shows that antibiotics were more commonly prescribed from the access category. Upper respiratory tract infections was the most common indication for which antibiotic was prescribed in primary health centres. In the tertiary care teaching hospital, half of the antibiotics were prescribed for cough, followed by pharyngitis (20 %).

Conclusions

A high proportion of antibiotics were prescribed for viral infections. Using antibiotics unnecessary can increase the cost of treatment as well as risk of antibiotic resistance. The Department of Medical Services, Puducherry should take initiative to ensure the successful implantation of the National Action Plan on Antimicrobial Resistance. Data of this study can be used to provide educational intervention for all drug stake holders such as physicians, pharmacists and policy makers to promote rational use of antibiotics.

## Introduction

Antimicrobial resistance (AMR) is a serious problem to solve for the public health authorities at the global level, particularly in developing countries like India. Worldwide deaths directly attributable to AMR are 700,000 per year, which is further projected to increase to 10 million by the year 2050 if current trends continue. The estimated cumulative loss of economic output from AMR by 2050 would amount to 20-35 trillion US dollars [1]. As per the report of the Centre of Disease Control, India, there is an increase in the risk of AMR for commonly seen diseases of public health concern, such as malaria, leprosy, meningococcal infections. kala-azar, TB & HIV [2]. India is one of the world's largest antibiotics consumers [3]. Antibiotic use in India doubled between 2000 (3.2 billion defined daily dose) and 2015 (6.5 billion defined daily dose) [4]. One of the possible reasons for AMR could be the inappropriate or overuse of antibiotics [5]. Published community studies have reported that for diseases like diarrhoea and fever which are primarily viral in nature, approximately 70% of patients in the healthcare facilities are given antibiotics [6]. In many countries of the SEA (South East Asia) region, antibiotics are freely available over the counter without prescription which is against regulations [7].

A multi-country survey conducted by the WHO (World Health Organization) revealed widespread public misunderstanding about antibiotic usage and resistance. The survey highlights for India are of concern and revealed that three quarters (75%) of respondents thought incorrectly that colds and flu can be treated with antibiotics. and only 58% knew that they should stop the antibiotics only when the course is completed as directed. More than three quarters (76%) of respondents reported taking antibiotics within the past 6 months and 90% said they were prescribed or provided by a doctor or nurse [8].

It has been observed that restricting misuse of antibiotics reduces resistance [9]. In 2017, WHO experts have revised the antibiotic section of EML (Essential Medicines List) where antibiotics are divided into three categories i.e. access, watch, and reserve. It provides advice on which antibiotics to choose while treating common bacterial infections, and which antibiotics to save for severe disease. The purpose of this modification is to ensure that antibiotics are available when needed and that the correct antibiotic is prescribed for appropriate infection [10].

India is coming forward to resolve the problem of AMR by implementing the National Action Plan on antimicrobial resistance (NAP-AMR) and improve the use of antibiotics by doctors, consumers and health institutions [11]. The Indian government implemented NAP-AMR in 2017. NAP-AMR emphasizes the need for improving the knowledge on rational use of antibiotics among various healthcare stakeholders through monitoring and research activities [12]. The plan set out five objectives i.e to improve awareness and understanding of antimicrobial resistance, to strengthen surveillance and research, to reduce the incidence of infection, to optimise the use of antimicrobials, and to ensure sustainable investment in countering antimicrobial resistance [13]. The Indian Medical Association (IMA) also has launched four campaigns for health care professionals to tackle this public health hazard - ‘Jarrurat bhi hai kya’ (meaning is there any need ?), "3A" meaning avoid antibiotic abuse’, ‘use wisely not widely' and 'think before ink”. The Indian Council of Medical Research (ICMR) has also promoted this plan by implementing an anti-microbial stewardship program at 20 private and public hospitals in the country [14].

Public health facilities remain the main source of health care services for the majority of the population. However, there is a paucity of studies regarding antibiotic use in public health facilities. Therefore, we conducted this study to monitor antibiotic use in public health facilities of Puducherry which is a union territory of south India. The data of this study will provide the scenario of antibiotic use in Puducherry public health facilities and could help to provide educational intervention for various stakeholders like prescribers, pharmacists, drug suppliers, and policymakers to promote rational use of antibiotics. 

## Materials and methods

A cross-sectional study was conducted to monitor antibiotic use in public health facilities of a union territory of South India. This study was conducted for a period of one year (from March 2019 to February 2020) in Puducherry. Pondicherry is a union territory of India and consists of four small districts: Puducherry, Karaikal, Yanam and Mahe. Puducherry district is the largest among the four and more representative of Pondicherry. Puducherry district has an adequate number of public health facilities functioning under both central (1 tertiary care hospital and 2 primary health centres) and state government (27 primary health centres, 2 community health centres and 5 district hospitals). A sample of 10 public health facilities was selected based on their geographical location. The sample included nine PHCs (primary health centres) and one tertiary care teaching hospital. These nine PHCs were selected randomly based on their geographical location (at least one PHC from North, South, West & East) and number of PHCs present in each direction (at least one from area with 1-5 PHCs, two from area with 5-10 PHCs and three from area with more than 10 PHCs). Thus, we selected a total of nine PHCs (one from north, three from south, three from west and two from east). Out of the nine PHCs, seven PHCs are functioning under state government and two PHCs are functioning under the central government. In the tertiary care hospital, the OPD (Outpatient Department) unit of the medicine department was included, as it is more representative of a PHC set up in India.

For antibiotics prescribing, we collected a total of 900 prescriptions. We included all patients visiting the outpatient departments of the selected health facilities. Pregnant women and patients with psychiatric illness were excluded from this study. To avoid seasonal bias, data were collected in three quarters (from March to June, July to October and November to February). In each quarter, 30 prescriptions were prospectively collected from each health facility to reach a total of 900 prescriptions in a year. Around 50-100 patients visit PHC on a single day. Therefore, 10 consecutive prescriptions were collected in the middle of the clinic day to reach the sample size of 10 per day for three days in a week to get 30 prescriptions per season from each health facility.

Data collection was done by qualified personnel having a Pharm.D (Doctor of Pharmacy) degree. Proper training was given for data collection prior to the field visit. A pilot study was also conducted to check the feasibility of data collection. Data were collected by taking photographs of prescriptions after taking informed consent from each participant. Data such as patient's demographic details (name, age, gender), diagnosis and medicines prescribed were entered in the data collection form. 

Data were entered in Microsoft Office Excel. Data were analysed separately for state govt. PHCs, central govt. PHCs and tertiary care teaching hospital. In the statistical analysis, frequencies, averages/means, and percentages were calculated. Statistical analysis was done by using Microsoft Office Excel 2010.

## Results

**Figure 1:**
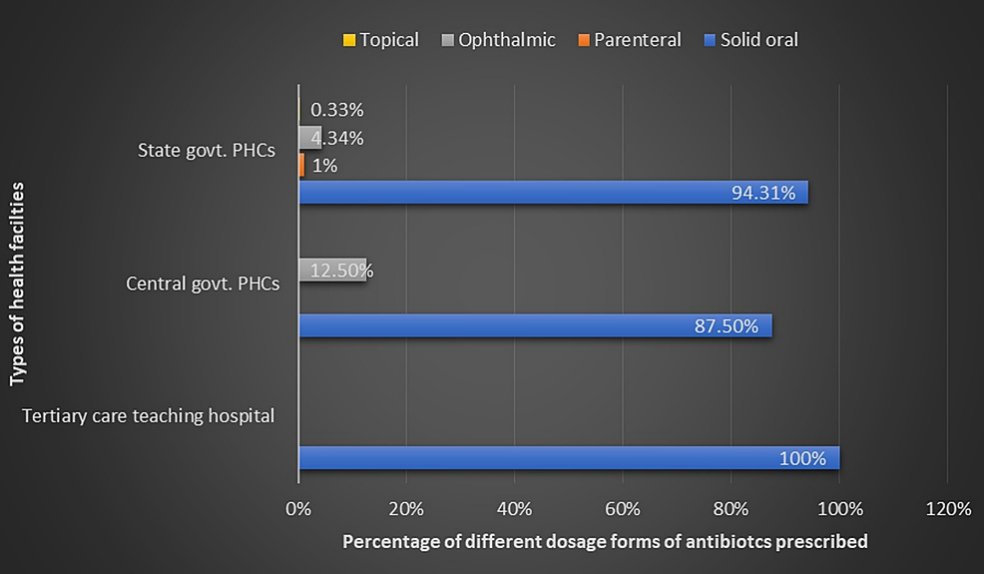
Percentage of different dose forms of antibiotics prescribed in surveyed public health facilities of Puducherry.

**Figure 2:**
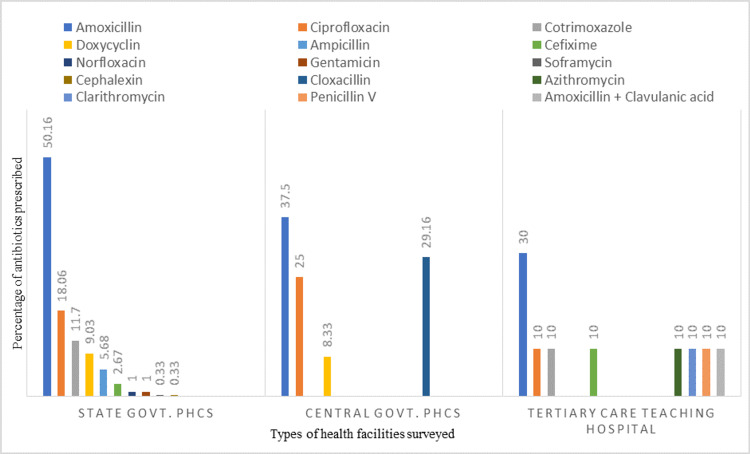
Different types of antibiotics prescribed in surveyed public health facilities of Puducherry

A total of 900 prescriptions i.e. 630 from state govt. PHCs (Primary Health Centres), 180 from central govt. PHCs and 90 from a tertiary care teaching hospital, were studied for antibiotic use. We found that 36.66 % of prescriptions (47.14 %, 13.33 % and 10 % in state govt. PHCs, central govt. PHCs and tertiary care teaching hospital respectively) contained at least one antibiotic. Some prescriptions had more than one antibiotic. Thus, the total number of antibiotics prescribed were 299 in state govt. PHCs, 24 in central govt. PHCs and 10 in tertiary care teaching hospital. Various dose forms of antibiotics prescribed and percentage of different types of antibiotics prescribed in surveyed health facilities is given in Figure 1, 2.

In our study, we found that antibiotics were prescribed more for males (60.8 %, 62.5 % and 60 % in state govt. PHCs, central govt. PHCs and the tertiary care teaching hospital respectively). In all three types of health facilities, antibiotics prescribing was highest for the age group 25-64 years. In all three types of health facilities, antibiotic use was reported more in the 2nd quarter (37.4 % in state govt. PHCs, 41.6 % in central govt. PHCs and 70 % in the tertiary care teaching hospital). The baseline characteristics of patients who were prescribed antibiotics are given in Table 1. 

**Table 1:** Basic characters of patients with antibiotics prescribed in surveyed public health facilities of Puducherry N= Number of health facilities surveyed; n = total number of antibiotics prescribed

Subgroups	State Govt. PHCs (N = 7) (n= 299)	Central Govt. PHCs (N = 2) ( n = 24)	Tertiary care teaching hospital (N = 1) (n = 10)
Age group (Years)			
14 years or less	19 (6.3 %)	3 (12.5 %)	0 (0 %)
14 – 24 years	59 (19.7 %)	2 (8.3 %)	0 (0 %)
25 – 64 years	195 (65.2 %)	15 (62.5 %)	9 (90 %)
65 years or more	26 (8.7%)	4 (16.6 %)	1 (10 %)
Gender			
Male	182 (60.8 %)	15 (62.5 %)	6 (60 %)
Female	117 (39.1 %)	9 (37.5 %)	4 (40 %)
Numbers (%) of antibiotics prescribed in three different quarters			
1^st^ quarter (March – June)	95 (31.7 %)	6 (25 %)	1 (10 %)
2^nd^ quarter (July – October)	112 (37.4 %)	10 (41.6 %)	7 (70 %)
3^rd^ quarter (November – February)	92 (30.7 %)	8 (33.3 %)	2 (20 %)

Our result shows that in all three types of health facilities, antibiotics were more commonly prescribed from the access category followed by the watch category, while none of the antibiotics were prescribed from the reserve category (Table 2).

**Table 2:** AWaRe (Access, Watch and Reserve) classification of antibiotics prescribed in surveyed public health facilities of Puducherry. N = Number of health facilities surveyed; n = number of antibiotics prescribed

AWaRe categories	State govt. PHCs ( N = 7; n = 299)	Central govt. PHCs (N = 2;n =24)	Tertiary care teaching hospital (N = 1; n = 10)
Access	Amoxicillin (50.1 %)	Amoxicillin (37.5 %)	Amoxicillin (30 %)
	Cotrimoxazole (11.7 %)	Cloxacillin (29.1 %)	Cotrimoxazole (10 %)
	Doxycycline (9 %)	Doxycycline (8.3 %)	Cloxacillin (10 %)
	Ampicillin (5.6 %)		Penicillin V (10 %)
	Gentamicine (1 %)		Amoxicillin + clavulanic acid (10 %)
	Cefalexine (0.3 %)		
Watch	Ciprofloxacin (18 %)	Ciprofloxacin (25 %)	Ciprofloxacin (10 %)
	Cefixime (2.6 %)		Cefixime (10 %)
	Norfloxacin (1%)		Azithromycin (10 %)
	Suframycine (0.3%)		
Reserve	Nil	Nil	Nil

**Figure 3:**
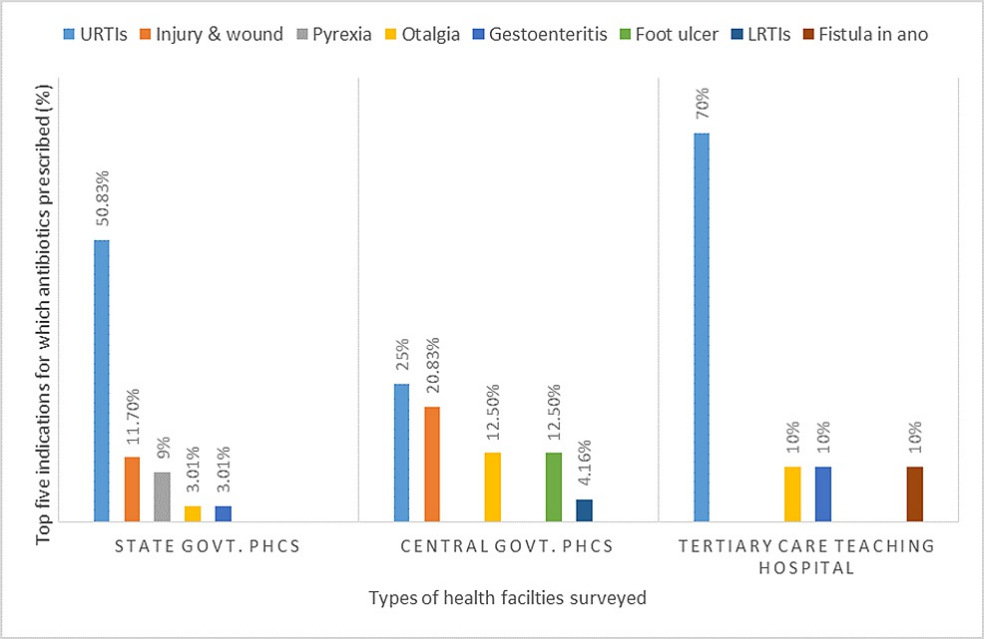
Top five indications for which antibiotics were prescribed in surveyed public health facilities of Puducherry URTIs: Upper Respiratory tract infections, LRTIs: Lower Respiratory Tract Infections

We found that URTIs (upper respiratory tract infections) was the most common indication for which antibiotic was prescribed in all surveyed health facilities. In state govt. PHCs, highest percentage of antibiotics (50.83%) were prescribed for URTIs such as common cold, pharyngitis and tonsillitis, followed by wound & injury (11.70%), and fever (9%). In central govt. PHCs, URTIs (25%), injury & wound (20.83%) and foot ulcer (12.3%) were top three indications for which antibiotics were prescribed. In the tertiary care teaching hospital, 70% antibiotics were prescribed for URTIs such as common cold, cough, pharyngitis followed by ear ache (10%), abdominal pain (10%) and fistula in ano (10%). Complete details of the top five indications for which antibiotics were prescribed in three types of health facilities are given in Figure 3.

## Discussion

In our study, antibiotic use was more in the 2nd quarter (July to October). Pondicherry has a tropical climate with no certain weather cycle. In Puducherry time, the period from July to September is mostly monsoon season. Light rain with cyclone formation continues till December. The monsoon season increases the risk of transmission of microbial diseases. A high incidence of bacterial and viral diseases, especially in rural areas in monsoon season, could be the reason for high antibiotic prescribing in the 2nd quarter (July to October) followed by the 3rd quarter (November to February). In our study, we found that antibiotic prescribing was more for male patients which could be because of several reasons such as males are more prone to be exposed to a polluted, contaminated environment, and communicate more with other people in day to day life which leads to increased risk of infections. In our study, we found that many male patients were prescribed antibiotics for cut injury and other accidental injuries which occurred at their job locations.

To overcome the global problem of AMR, the WHO categorized antibiotics into three categories i.e. access, watch, and reserve, with the aim to increase treatment effectiveness, minimise drug resistance development, and preserve the effectiveness of "last resort" antibiotics [15]. In our study, we found that in all three types of health facilities, most of the antibiotics prescribed were from the access category, while few were from the watch category. and none of antibioticss were prescribed from the reserve category. The high rate of prescribing access antibiotics in our study could be due to the availability of these access antibiotics in surveyed health facilities. Few antibiotics are prescribed from the watch category, which could be due to the lack of availability of access antibiotics for particular infections, or lack of awareness regarding WHO AWaRe among clinicians of Puducherry public health facilities. Our results indicate the need for creating awareness about WHO’s AWaRe strategy among prescribers, and also to maintain a regular supply of access antibiotics in public health facilities.

Antibiotics are available in different dosage forms and should be given according to the requirement of the patient. However, solid oral dosage forms such as capsules and tablets should be given preference, especially in adult patients because of advantages such as easy to swallow, high precision, lowest variability, more stability, accurate dosing, and easy transportation. In our study, most of the antibiotics (94.31 in state govt. PHCs, 87.5% in central govt. PHCs, and 100% in tertiary care teaching hospital) were prescribed as oral solid dosage form which is in agreement with the finding (94.8%) of a study conducted in Addis Ababa, Ethiopia [16]. Our findings are higher than findings reported in the study conducted in a hospital in Northeast Ethiopia where 58% of the antibiotics are prescribed by oral route [17]. A high prescribing of solid dosage forms could be because of more adult patients, prescriber’s awareness about the advantages of solid oral dosage forms, and the availability of appropriate dosage forms of prescribed antibiotics. In our study, we found that a few antibiotics were prescribed in form of creams which is for dermatological bacterial infections such as cellulitis. In our study, we found that a few antibiotics were prescribed in the form of drops for treating ear and eye infections, which is as per recommended guidelines. 

In our study, we found that amoxicillin was the most commonly prescribed antibiotic, followed by ciprofloxacin and cotrimoxazole. Our findings are in agreement with studies conducted in China [18] and Nigeria [19] where amoxicillin was the most frequently prescribed antibiotic and accounted for 21.3% and 25.4% of the total prescribed antibiotics, respectively. Three studies conducted in Ethiopia were also reported Amoxicillin as the most commonly prescribed antibiotic [16,20,21]. A study conducted in Turkey also reported that Amoxicillin/clavulanic acid was the most commonly used antibiotic (18.1%) [22].

Our results indicate that a high percentage of antibiotics were prescribed for viral infections and fever. In the current study, we found that the most common indication for which antibiotic prescribed was URTIs (50.83% and 25% in state and in central govt. PHCs respectively), which is in agreement with the study conducted in Adis Ababa, Ethiopia where the most common diagnosis for antibiotic use was URTIs (24.5%) [16]. The current study is also in agreement with studies conducted in Malaysia [23] and Yemen [24], where the most common indication for antibiotics prescribing was URTIs and accounting for 49.2% and 38% of the cases, respectively. Antibiotics are not recommended for viral infections, and it is important to differentiate between bacterial and viral infections to avoid irrational or overuse of antibiotics. URTI is considered a viral-originated infection and 70-80 % of URTIs are because of viruses. Studies reported that prescribing antibiotics in URTIs is very common and 70-80% of patients with URTIs worldwide are being prescribed antibiotics [25]. Prescribing antibiotics in viral infections not only increases the risk of antibiotic resistance, but also increases the cost of therapy. A study conducted in Turkey reported that antibiotics accounted for half (50.2%) of the total cost of medicines prescribed [22]. Avoiding the use of antibiotics for viral infections can significantly reduce the cost of treatment and also minimize the risk of development of antibiotic resistance.

Our study has certain limitations. We conducted this study only in public health facilities. Thus, our results may not reflect the quantum of antibiotics use/misuse in private health facilities. The appropriateness of antibiotic use could not be confirmed by microbiological testing in patients who were prescribed antibiotics due to the lack of facilities at the PHC level. 

## Conclusions

We report a high rate of antibiotic prescribing, especially at primary health centres. A high proportion of antibiotics were prescribed for conditions like URTIs such as common cold, pharyngitis, and tonsillitis which are viral in origin. Prescribing antibiotics for viral infections may increase the rate of AMR development as well as the cost of treatment for both patients and the government. Our study indicates that India’s NAP-AMR is not being implemented and followed in Puducherry. The Department of Medical Services, Puducherry should take initiatives to ensure the successful implantation of NAP-AMR in Puducherry. There is also a need to establish a separate committee to monitor and control the irrational use of antibiotics in Puducherry. Providing educational intervention for all drug stakeholders such as physicians, pharmacists, and policymakers could help to improve the rational use of antibiotics.
